# Clival ectopic pituitary prolactinoma was successfully managed by transsphenoidal surgery: A rare case report

**DOI:** 10.1002/ccr3.8255

**Published:** 2023-11-20

**Authors:** Marah Mansour, Zeinah Khozamah, Abdulmonem Naksho, Roaa Zayat, Aliaa Al Sabbagh, Ahmad Deeb

**Affiliations:** ^1^ Faculty of Medicine Tartous University Tartous Syria; ^2^ Division of Colon and Rectal Surgery, Department of Surgery Mayo Clinic Hospital Rochester Minnesota USA; ^3^ Department of ENT Ibn Al‐Nafees Hospital Damascus Syria; ^4^ MSc Global Public Health Nutrition, School of Life Sciences University of Westminster London UK; ^5^ Doctor of Medicine, Faculty of Medicine AlBaath University Homs Syria; ^6^ Faculty of Medicine University of Damascus Damascus Syria; ^7^ Department of ENT Al Basel Hospital Syria

**Keywords:** case report, clival tumors, ectopic pituitary adenoma, prolactinoma, stalk effect, transsphenoidal surgery

## Abstract

**Key Clinical Message:**

Ectopic pituitary adenoma is a rare neoplasm located in the clivus and could mimic other clival tumors. Diagnosis and treatment could be challenging. It should be considered in the differential diagnosis of clival tumors.

**Abstract:**

Ectopic pituitary adenomas (EPAs) are isolated adenomas that can be located in variable locations outside the sella turcica and have a normal‐appearing pituitary gland. These tumors are rare and are thought to often arise from embryological remnants along the route of Rathke's pouch migration. EPAs are associated with a wide range of clinical manifestations depending on hormonal activity and involvement of adjacent structures, which can represent a challenge in making the diagnosis and deciding on the most appropriate management. In this case study, we report a 47‐year‐old male who presented with visual disturbances, a headache, and generalized weakness. Magnetic resonance imaging showed a 2 cm mass located in the clivus invading the sphenoid sinus with an intact pituitary gland. The patient underwent endoscopic transsphenoidal surgery to eradicate the mass while maintaining the integrity of the pituitary gland, which was successful and uneventful. Pathological studies were consistent with prolactinoma, with no cytological malignant features. Post‐surgery, symptoms notably improved, and serum prolactin levels significantly dropped, The patient's condition was satisfactory on follow‐up with no long‐term complications reported. This paper contributes to the existing literature by sharing the clinical management of a challenging and uncommon case.

## Graphical abstract

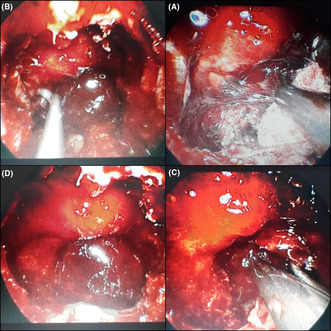

## 1 INTRODUCTION

Ectopic pituitary adenomas (EPAs) are isolated adenomas located outside the sella turcica with a normal intrasellar pituitary gland. EPAs are thought to be extremely rare tumors.^1^ The location of EPAs can vary, with approximately 60% of reported cases found in the sphenoid sinus and suprasellar region, 30% in the clivus, nasal cavity, parasellar region, cavernous sinus, and sphenoid wing.^2^ The origin of EPAs can take one of three forms, with the most common being adenomas derived from residual cells of Rathke's pouch, which persist along the developmental pathway of the anterior pituitary gland. Clinical manifestations of EPAs usually depend on hormonal activity and the involvement of adjacent structures. For example, tumors extending into the cavernous sinus or clivus can compress cranial nerves (CNs), leading to visual disturbances and facial paresthesia, while sphenoid sinus EPA can result in headaches, nasal obstruction and cerebrospinal fluid (CSF) leaks.^2^ The diagnosis and management of EPAs can be challenging and usually require strong clinical suspicion along with magnetic resonance imaging (MRI) findings of a normal pituitary gland, which are confirmed by pathological studies. Computerized tomography (CT) can also assist in determining the invasion of the sphenoid sinus, aiding in the planning of endoscopic trans‐sphenodial surgery, which is considered the most appropriate management approach.^3^, ^4^ In this case, we present a clival ectopic pituitary prolactinoma.

## 2 CASE PRESENTATION

**FIGURE 1:**
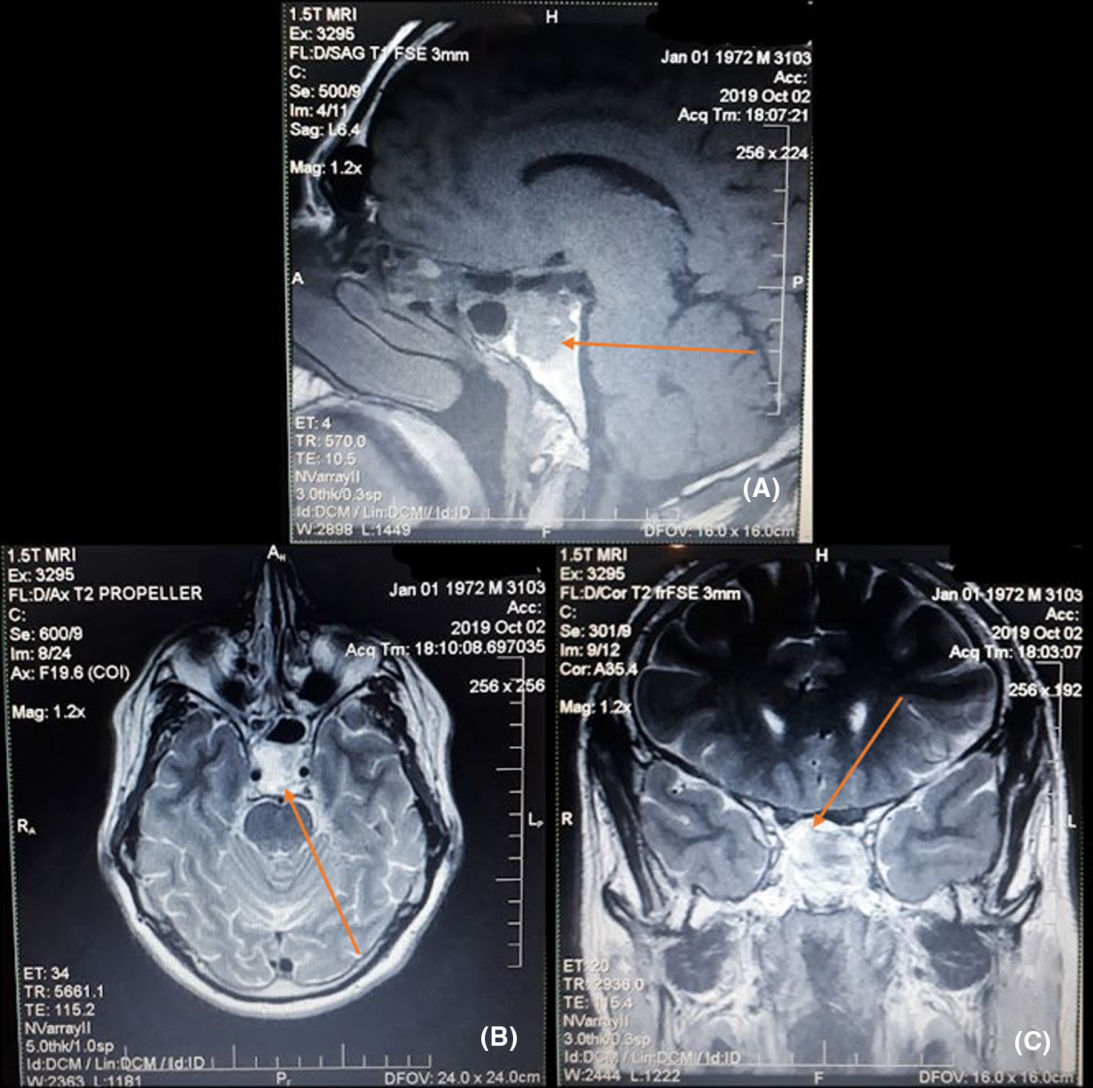
(A, B, and C): MRI with contrast of sphenoid sinus and pituitary gland in sagittal (A), transverse (B), and coronary (C) sections showing a mass in the clivus with moderate enhancement and peripheral calcifications.

**FIGURE 2:**
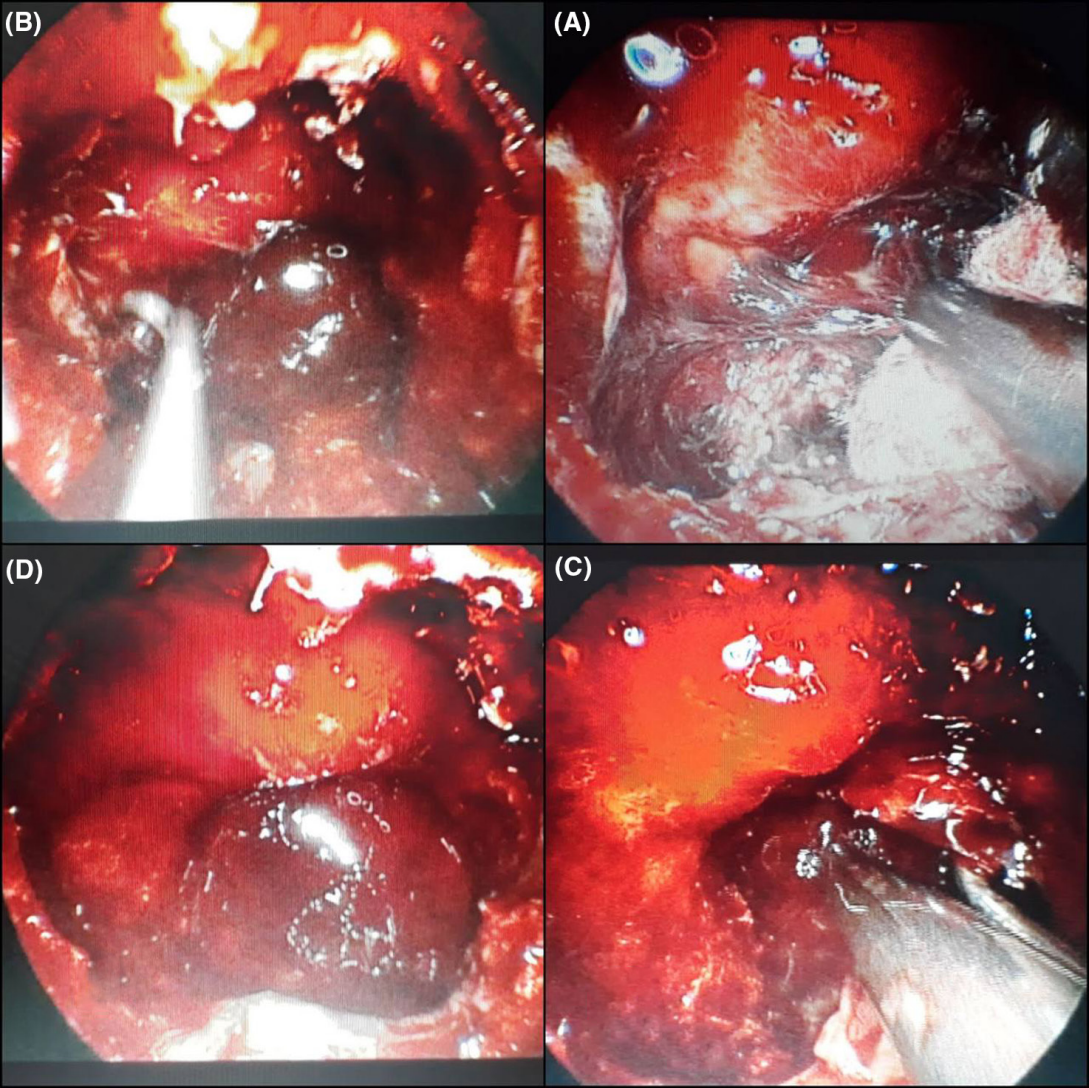
(A, B, C, and D): Endoscopic images of transsphenoidal surgery showing the neoplastic mass adjacent to the intact pituitary gland (A), the neoplastic mass adjacent to both the right and left ICAs (B, C), and the pituitary gland after complete resection of the mass (D).

**FIGURE 3:**
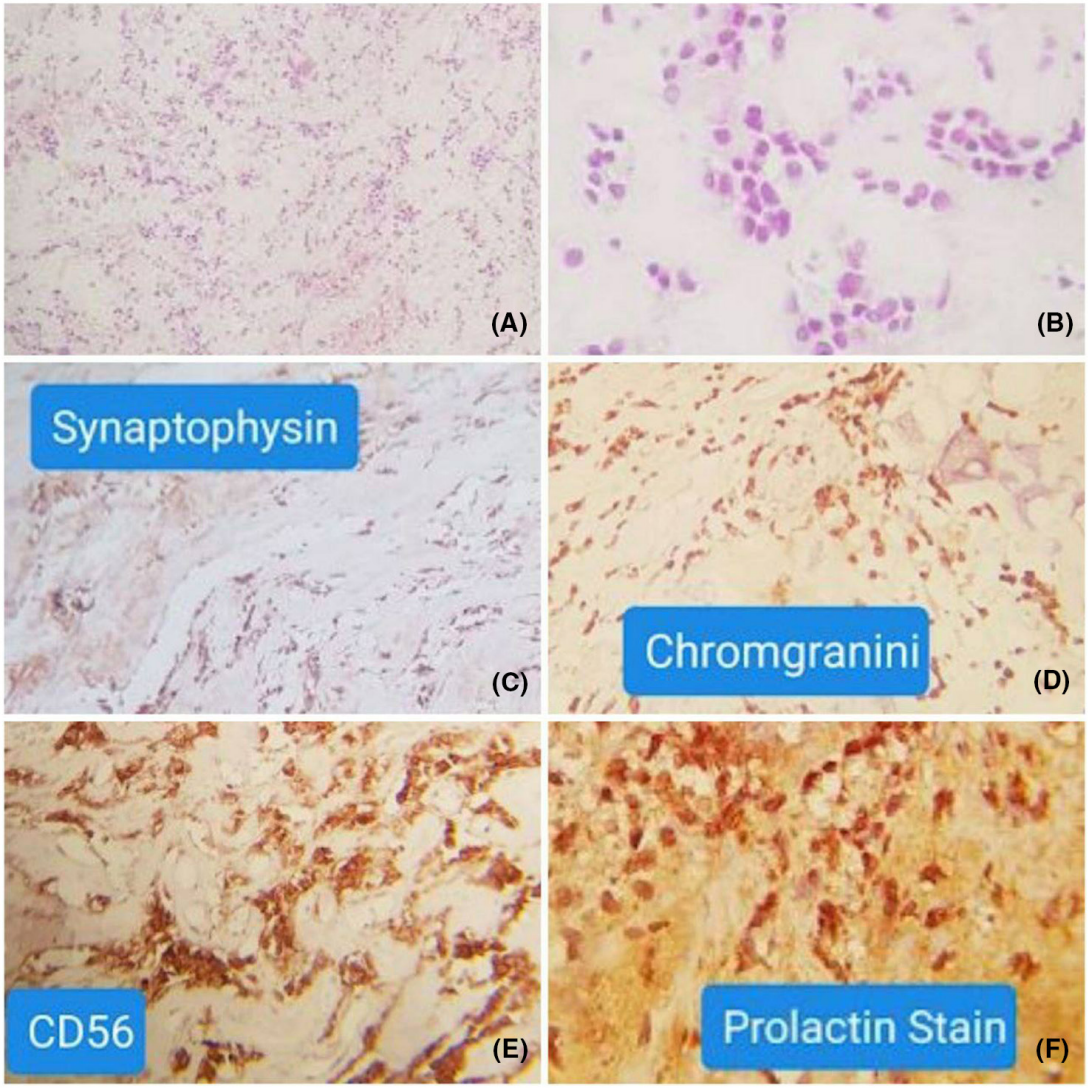
(A, B, C, D, E, and F): Histological images of the resected mass showing small nests of uniform small oval‐to‐polygonal‐shaped cells surrounded by dense hyaline stroma (A, B), negative staining for synaptophysin, chromogranin, and CD56, respectively (C, D, E), and positive staining for prolactin (F).

A 47‐year‐old male presented to the ENT Department (Ear, Nose, and Throat) with a 1‐year history of both parietal and occipital headaches, retro‐orbital pain, transient blurry vision, fatigue, and difficulty completing routine tasks. However, there were no reported symptoms of hyperprolactinemia. Family, medical, surgical, and drug histories were unremarkable. The physical examination was insignificant. However, an optic nerve examination was not performed before the surgery. Laboratory findings were normal, except for elevated serum prolactin of 269 ng/mL. MRI, with contrast of the sphenoid sinus and pituitary gland, revealed a 2 cm mass within the clivus, invading the sphenoid sinus. The mass showed moderate enhancement with peripheral calcifications. The pituitary gland and its stalk appeared to be normal, in Figure 1A–C. The mass was totally resected via trans‐sphenoidal endoscopic surgery, no medications were administered prior to the procedure. The posterior portion of the nasal septum was resected, and the inlet was expanded while maintaining the integrity of the sphenopalatine artery. The intersphenoid septum was removed to reach the posterior wall of the sphenoid, which was invaded by the tumor. However, the pituitary gland was notably intact. The neoplastic mass looked fibrous, and it was carefully resected without any vascular or neurologic complications. The internal carotid arteries, CNs III/IV/V/VI, and the optic chiasm were all maintained, in Figure 2A–D. The postoperative period was uneventful. Gross examination revealed fragments of tan, white tissue measuring about 1.2 × 1 × 0.5 cm with bony chips. Microscopic examination showed a loosely infiltrating tumor composed of irregular cords and small nests of uniformly small oval‐to‐polygonal‐shaped cells surrounded by dense hyaline stroma and bone. In isolated areas, dystrophic calcifications were noted. Immunostains were performed for prolactin, Ki‐67, CD138, Epithelial Membrane Antigen, CD45, CD34, S‐100, protein, synaptophysin, chromogranin, and CD56. The tumor cells were strongly positive for prolactin, while the others were negative. No cytological malignant features were noted, in Figure 3A–F. Postoperatively, the patient recovered rapidly; his symptoms notably improved, and the serum prolactin level dropped to 26 ng/mL. On an 18‐month follow‐up, the patient's condition was satisfactory, with no long‐term complications.

## 3 DISCUSSION AND CONCLUSIONS

EPAs are rare extracellular pituitary adenomas that have no correlation with the components located inside the sella turcica. They are often found along the migration pathway of Rathke's pouch; however, the exact pathogenesis remains unclear.^3^, ^4^ Unlike our case, where the patient is a male, EPAs tend to present more frequently in females, most frequently between the fourth and seventh decades of life, with the mean age at diagnosis being 51.4 years old.^3^ In this case, we report an EPA located in the clivus, representing the third most common location for EPAs, after the sphenoid sinus and suprasellar region.^4^ Establishing the diagnosis of EPAs can be challenging as presentation varies widely depending on several factors, including anatomy, hormonal activity, and involvement/invasion of surrounding structures.^2^ When considering the differential diagnosis (DD), the clivus can be affected by a number of conditions, including lesions arising within the clivus or from adjacent structures, with chordoma being the most common tumor of this region, which can usually be differentiated from EPAs on imaging inspection. However, a biopsy is usually required to confirm the diagnosis.^4^, ^5^ Other DDs include intraosseous meningioma, chondrosarcoma, solitary plasmacytomas, myelomas, astrocytomas, craniopharyngioma, germ cell tumor, non‐Hodgkin's lymphoma, melanoma, and metastases.^1^, ^4^ The stalk effect due to a nonfunctional adenoma should be considered in DD with elevated prolactin levels. However, prolactin levels >200–250 ng/mL are suspicious of prolactinomas, whereas prolactin levels resulting from pituitary stalk compression are generally <100 ng/mL and rarely exceed 250 ng/mL.^6^ Consistent with the presenting complaints in our report, clival EPAs, similar to other clival lesions, are known to exhibit mass‐effect symptoms such as headache and focal cranial nerve palsy.^5^, ^7^ Fifty‐eight percent of EPAs are functional tumors. In some clival diseases, endocrinopathy may result from the infiltration of the pituitary. Clival EPAs can invade adjacent structures, causing destructive features such as bone erosion, in keeping with the tumor in our case, which invaded the sphenoid sinus.^4^, ^8^, ^9^, ^10^ Due to the complexity of EPAs, several factors should be considered for treatment, including tumor size and location, clinical manifestations, hormone‐secreting type, and extent of invasion.^4^ The majority of EPAs are definitively diagnosed following surgery and histopathological examination.^11^ Surgery should be considered in pituitary prolactinomas when: the patient cannot tolerate medical therapy, medical therapy is ineffective, there are macroadenomas with a cystic component, or there is optic nerve compression.^12^ However, ectopic prolactin‐secreting adenoma's management remains controversial between medical and surgical.^13^ The transsphenoidal approach is an excellent method for accessing clival tumors, offering favorable exposure for surgical intervention.^14^

Herein, trans‐sphenoidal endoscopic surgery was performed as a diagnostic and therapeutic procedure in our case. Compared with other locations (cavernous sinus, suprasellar region, and sphenoid sinus), the clival EPAs are relatively more difficult to reach, detect, and confirm with an endoscopic biopsy. In addition, experience in EPA treatment is often lacking, considering how rare these tumors are.^4^ In conclusion, the diagnosis of clival ectopic pituitary prolactinomas may be difficult. However, a histological study is necessary to confirm the diagnosis, especially when there are no hormonal‐related symptoms. Our case represents a significant contribution to the existing medical literature on ectopic prolactinoma as a rare case, considering that an increased number of published cases will enhance knowledge and lead to more accurate statistical analysis.

## AUTHOR CONTRIBUTIONS

**Marah Mansour:** Conceptualization; data curation; formal analysis; funding acquisition; investigation; methodology; project administration; resources; software; supervision; validation; visualization; writing – original draft; writing – review and editing. **Zeinah Khozamah:** Conceptualization; data curation; formal analysis; investigation; methodology; resources; software; writing – original draft; writing – review and editing. **Abdulmonem Naksho:** Conceptualization; data curation; formal analysis; investigation; methodology; resources; software; writing – original draft; writing – review and editing. **Roaa Zayat:** Data curation; investigation; resources; software; writing – original draft; writing – review and editing. **Aliaa Al Sabbagh:** Conceptualization; data curation; investigation; resources; software. **Ahmad Deeb:** Conceptualization; data curation; funding acquisition; investigation; methodology; project administration; resources; software; supervision; validation; visualization.

## FUNDING INFORMATION

No funding was required.

## CONFLICT OF INTEREST STATEMENT

The authors declare that they have no conflicts of interest.

## ETHICS STATEMENT

Not required for this case report.

## CONSENT

Written informed consent was obtained from the patient for publishing this case report and any accompanying and identifying images or other personal or clinical details of this patient that compromise anonymity. A copy of the written consent is available for review by the Editor‐in‐Chief of this journal on request.

## GUARANTOR

Ahmad Deeb is the guarantor of this work.

## Data Availability

Not applicable. All data (of the patient) generated during this study are included in this published article and its supplementary information files.
